# Learning-Induced Improvement in Encoding and Decoding of Specific Movement Directions by Neurons in the Primary Motor Cortex

**DOI:** 10.1371/journal.pbio.0020045

**Published:** 2004-02-17

**Authors:** Rony Paz, Eilon Vaadia

**Affiliations:** **1**Interdisciplinary Center for Neural Computation, The Hebrew UniversityJerusalemIsrael; **2**Department of Physiology, The Hebrew University-Hadassah Medical SchoolJerusalemIsrael

## Abstract

Many recent studies describe learning-related changes in sensory and motor areas, but few have directly probed for improvement in neuronal coding after learning. We used information theory to analyze single-cell activity from the primary motor cortex of monkeys, before and after learning a local rotational visuomotor task. We show that after learning, neurons in the primary motor cortex conveyed more information about the direction of movement and did so with relation to their directional sensitivity. Similar to recent findings in sensory systems, this specific improvement in encoding is correlated with an increase in the slope of the neurons' tuning curve. We further demonstrate that the improved information after learning enables a more accurate reconstruction of movement direction from neuronal populations. Our results suggest that similar mechanisms govern learning in sensory and motor areas and provide further evidence for a tight relationship between the locality of learning and the properties of neurons; namely, cells only show plasticity if their preferred direction is near the training one. The results also suggest that simple learning tasks can enhance the performance of brain–machine interfaces.

## Abstract 2

Information theory is used to analyze neuronal responses in monkeys trained to direct movements to a visual target

## Introduction

Practice can induce behavioral improvement that is often specific to the situation experienced during the practice sessions (or “training”). Such findings suggest that changes occur in neurons with fine selectivity (or “tuning”) for the stimuli experienced or the movements made during training. In the visual system, for example, behavioral improvement is specific to the trained stimulus, such as the orientation of a light bar (Fiorentini and Berardi 1980; Crist et al. 1997), and is paralleled by specific changes in neurons that are tuned to the orientation of a light bar (Schoups et al. 2001) or, in other experiments, the direction of visual motion (Zohary et al. 1994). In the auditory system, changes in response properties of single neurons and cochleotopic maps are specific to the parameters characterizing the sound (Suga et al. 2002). In the motor system, skill acquisition induces expansion in the cortical representation of the used forelimb (Nudo et al. 1996) and enhance synaptic connections in the trained contralateral hemisphere (Rioult-Pedotti et al. 2000). A line of studies found that when monkeys perform reaching movements and adapt to directional errors induced by force fields, primary motor cortex (M1) cells shift their preferred direction (PD) in about the same way as for the muscle activity needed to perform the task (Gandolfo et al. 2000; Li et al. 2001; Padoa-Schioppa et al. 2002). We have recently shown that learning a local rotational visuomotor task can induce an elevation in the activity of single neurons in M1 (Paz et al. 2003) and that these changes are observed only in a specific subpopulation of neurons, those with a PD close to the movement direction used during the learning.

Whereas many studies indicate that learning can induce specific changes in brain activity, this finding does not necessarily imply that newly learned skills are “better” represented in the brain. The crucial question is this: Do neurons encode task parameters, such as movement direction, any better after learning? In the motor system, such improved encoding (Chen and Wise 1997) can be used for decoding by downstream areas and as an efference copy for further computation (Wolpert and Ghahramani 2000; Sommer and Wurtz 2002). It can also be used by an external observer to allow for more accurate prediction of behavior (Laubach et al. 2000). In this paper, we examine two questions. First, do learning-induced changes in firing rates provide more information on the task? And, second, what aspect of the cells' activity contributes mostly to this improvement?

**Figure 2:**
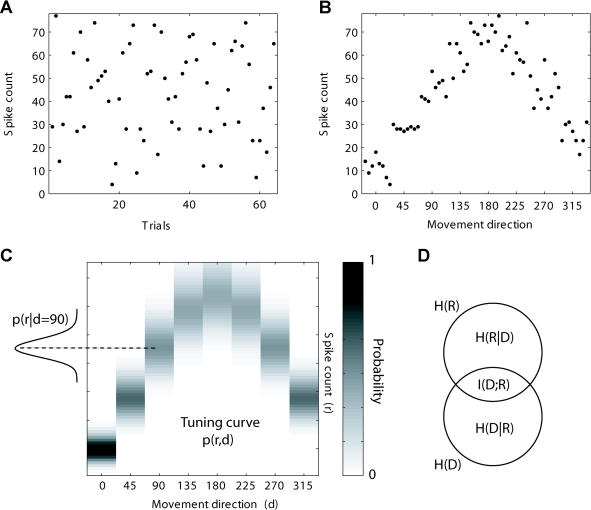
MuI between Neuronal Activity and Direction of Movement The example shows a simulation of the activity of one cell during 64 movements to evenly spaced eight directions, presented in a random order (eight trials per direction). Each dot in the raster plots a and b describes the spike count of the cell in a specific trial. Without prior knowledge about the direction of movement (A), a large uncertainty exists about the responses of the neuron. However, ordering the trials according to the movement direction (B) reveals a large reduction in the uncertainty about the cell responses. The probability p(*r,d*) of observing a trial with direction *d* and spike count *r* is shown in (C); along with a specific conditional distribution *p*(*r*|*d*_0 = 90)_ . The entropy 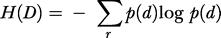 is a measure of the uncertainty about movement direction: H(D) = log(8) = 3 bits, in the case that all eight directions have equal probability to occur. The conditional entropy is defined as 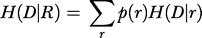 and describes the mean uncertainty about direction given the cell response. The MuI *I*(*R*;*D*) = *H*(*D*) − *H*(*D*|*R*) measures the reduction in uncertainty about movement direction given the response of the cell. The MuI is symmetric, in the sense that it also measures the reduction in uncertainty about cell response given the direction of movement *I*(*R*;*D*) = *H*(*D*) − *H*(*D*|*R*) . This relation is graphically depicted in (D).

To address the first question, we employed an information-theory analysis (Cover and Thomas 1991; Rieke et al. 1997) to calculate the mutual information (MuI) (see Figure 2) between cells' activity and direction of movement. Informational measures have two relevant advantages. First, they use the full distribution (estimated from the data) of neuronal activity and do not assume any specific shape of the tuning curve or noise distribution. This allows for a more fine-tuned examination of learning-related changes. Second, they provide a measure as to how well different directions can be differentiated, based on neuronal activity. To address the second question, we examined two features of the neuronal response that could contribute to the increase in information: response variability and the slope of the tuning curve. Finally, to demonstrate that the observed increase in information can be extracted, we use the neuronal activity to decode the actual movement direction.

## Results

**Figure 1:**
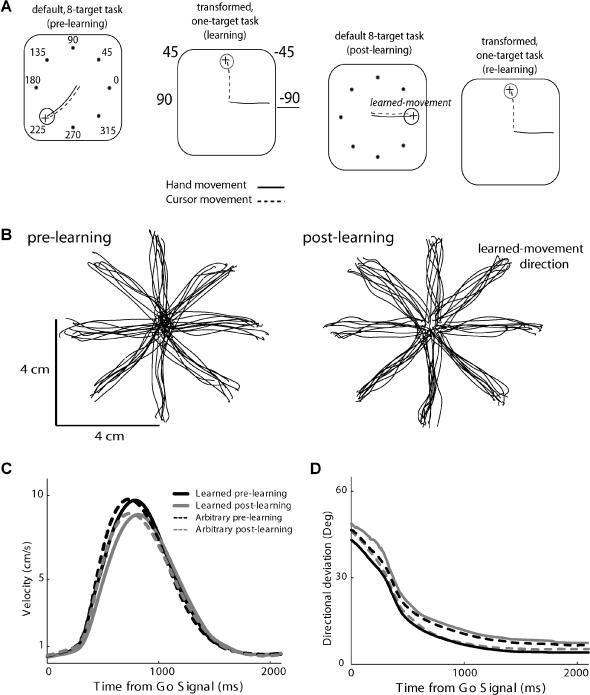
Behavioral Paradigm and Movement Kinematics (A) Session flow (left to right). Every session (day) consisted of pre-learning, learning, post-learning, and relearning epochs. Pre- and post-learning epochs were standard eight-target tasks with a default (one-to-one) mapping between cursor movement and the movement of the hand. In the learning epoch, only one target (upwards) appeared, and a visuomotor rotational transformation was imposed on the relationship between movement of the hand and cursor movement. The example shown is for a transform of −90° (seeMaterials and Methods for a full description). (B–D) Similar kinematics pre- and post-learning. (B) Example of 1-day trajectories from the two epochs; the transform in this session was of –45°. (C) Velocity profiles. Peak velocity was slightly lower in the post-learning epoch (*t*-test, *p* = 0.05), but no difference was found between the learned direction and other directions (*t*-test, *p* = 0.3). (D) Improvement in directional deviation was calculated as the deviation of the instantaneous hand direction from the required target direction, calculated every 10 ms starting from the go-signal. All four movement types (learned and nonlearned, pre- and post-learning) exhibited the same temporal pattern. Here and for analysis of neuronal activity, we excluded the first trials in the post-learning epoch—those exhibiting significant aftereffects due to learning.

Monkeys adapted to visuomotor rotations on a daily basis by altering the relationship between the visual feedback (cursor) and the hand movement (Figure 1). Learning was confined to only one target in space, i.e., learning that is local in direction. We tested neuronal sensitivity to direction by comparing the information content conveyed in the firing rate of single cells during the pre- and post-learning epochs (identical task of standard movements to eight directions spanning the two-dimensional working surface, only differentiated by a learning epoch). We specifically looked for a change in representation that was related selectively to the learned direction, i.e., the hand direction that was used to bring the cursor to the target during the transformation.

Activity was measured from the hold period that immediately follows the target appearance, but before the go-signal, and was therefore termed preparatory activity (PA). There were three reasons for this choice. First, such PA has been reported in many motor cortices and is thought to participate in movement planning and in computing visuomotor transformations (Kurata and Wise 1988; Alexander and Crutcher 1990; Kalaska et al. 1997; Shen and Alexander 1997; Zhang et al. 1997; Kakei et al. 2001). Second, as previously found in this experimental paradigm, learning-related changes have only been reported for this period (Paz et al. 2003). Third, as a means of eliminating any kinematic-related changes (Wise et al. 1998), we further verified that movements shared similar kinematics before and after learning (see Materials and Methods; Figure 1).

### Mutual Information

**Figure 3:**
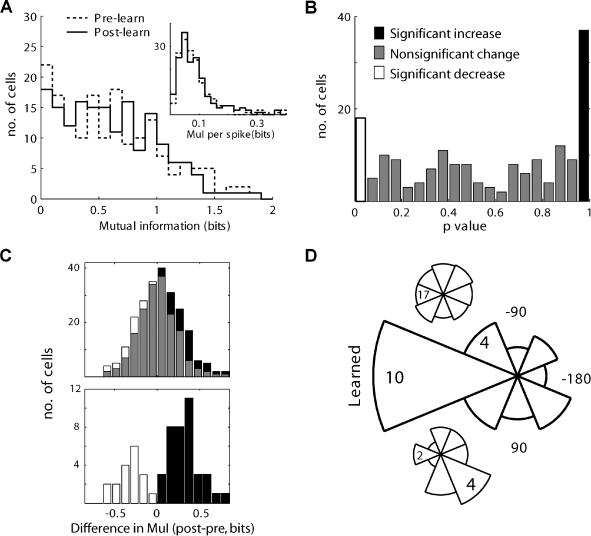
Comparing MuI of Single Cells Pre- and Post-Learning (A) Distributions of single-cell information about direction of movement pre-learning (dashed) and post-learning (solid). No significant difference was found between the distributions (Kolmogorov–Smirnoff, *p* = 0.3). The inset shows the MuI per spike, calculated by dividing the information per cell by the cell's firing rate (Kolmogorov–Smirnoff, *p* = 0.25). (B) Improvement in information of individual cells. Histogram of *p*-values for all cells; a significant (*p* < 0.01, χ^2^) number of cells (*n* = 37) had a *p*-value greater than 0.95, representing cells that significantly increase their information content about direction after learning; 18 cells had a *p*-value lower than 0.05, representing cells that decreased their information content, but this was found to be only marginally significant (*p* = 0.06, χ^2^). (C) Histograms of difference in information, post- minus pre-learning, for all cells (upper) and only for cells that increase (*p* > 0.95) or decrease (*p* < 0.05) their information content significantly (lower). (D) Circular histogram for PD of cells that significantly increased their information. The cells' PDs were normalized to the learned direction in each cell recording session, revealing a unimodal distribution (Rayleigh test, *p* < 0.05). The upper inset shows the circular histogram for all cells and lower inset shows the circular histogram for cells that decreased their information; in both cases, the distributions seem homogenous (Rayleigh test, *p* > 0.1).

The MuI between one-cell activity and direction of movement is exemplified in Figure 2. We compared the MuI between pre- and post-learning (Figure 3A). The figure depicts the distributions of MuI between direction and spike count for all cells (Figure 3A, corrected for bias) for pre-learning (dashed line) and for post-learning (solid line). No difference was found between the MuI on the population level, either by comparing the distributions (Kolmogorov–Smirnoff, *p* = 0.3) or by comparing their means (paired *t*-test, *p* = 0.53). We further tested the average information about direction conveyed by each spike by normalizing each cell's information by its firing rate and again found no significant difference (inset in Figure 3A; Kolmogorov–Smirnoff, *p* = 0.25, paired *t*-test, *p* = 0.7).

Although the population as a whole did not change significantly, single neurons could still increase or decrease their information about direction. To explore this, we probed each neuron individually for changes in MuI. Using a bootstrap method, we shuffled trials from pre- and post-learning and randomly reselected two different groups of trials, we then calculated the MuI for each group and the difference between the two MuIs. The procedure was repeated 1,000 times to produce a distribution of MuI differences. The actual MuI difference (between the pre- and post-learning) was compared to this distribution to obtain a *p*-value. A high *p*-value means that the MuI in the post-learning epoch was significantly higher than the MuI in the pre-learning epoch. Figure 3B plots the histogram of the *p*-values for all cells. A significant number of cells showed an increase in MuI with a *p*-value larger than 0.95 (black in Figure 3B; *n* = 37 out of 177, *p* < 0.01, χ^2^), a nearly significant number of cells showed a decrease in MuI with a *p*-value lower than 0.05 (white/transparent in Figure 3B; *n* = 18, *p* = 0.06), while all the rest did not (gray in Figure 3B). We also examined the actual change in information content for all cells (Figure 3C, upper) and specifically for the cells that had a significant change (Figure 3C, lower).

Following the rationale explained in the Introduction, the association between the learned parameter (direction) in local rotational transformations and the sensitivity of many cells to direction, we probed for a relation between cells' PD and the learned direction. Figure 3D plots a circular histogram of PDs of cells that exhibited a significant increase in their MuI. For the plot, we normalized each cell's PD to the learned direction in its recording session, and this revealed a unimodal distribution (Rayleigh test, *p* < 0.05) with its center on the learned direction. In contrast, the PD distributions of the whole population (Figure 3D, upper inset) and of cells that significantly decrease their information content (Figure 3D, lower inset) did not exhibit this trend and seemed homogenous.

**Figure 4:**
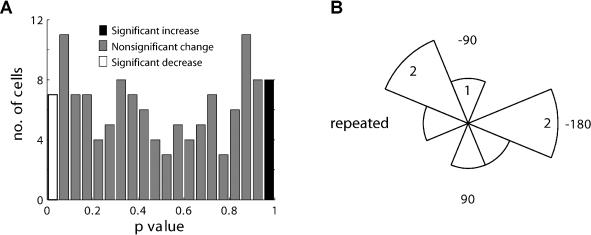
Changes Were Not Observed after Mere Repetition of Movement to One Direction Same as in Figure 3B and 3D, but for control sessions that included the mere repetition of standard, nontransformed movement to one target during the learning epoch. The number of cells that exhibited an increase in their information content was not significant ([A] right bar, eight out of 126), and their distribution was homogenous and showed no specific relation to the direction of the repeated movement (B).

To test that this change in information is indeed owing to the learning of visuomotor transformations and not owing to the mere repetition of a single movement during the learning epoch, we conducted the same analysis for control, repetition sessions. Only a nonsignificant (*p* > 0.1, χ^2^) number of cells (eight out of 126) had a *p*-value greater than 0.95 (Figure 4A). Further, this population did not exhibit any specific distribution of PDs (Figure 4B; Rayleigh test, *p* > 0.1).

### Individual Information per Direction

The MuI represents the information that a cell's spike count conveys about all the eight tested directions. We further investigated how much information a cell conveys about one direction in particular, which is termed the individual information per direction (DI) (Rolls et al. 1997; Buracas et al. 1998) and is measured as the reduction in uncertainty about the spike counts, given a specific direction.

**Figure 5:**
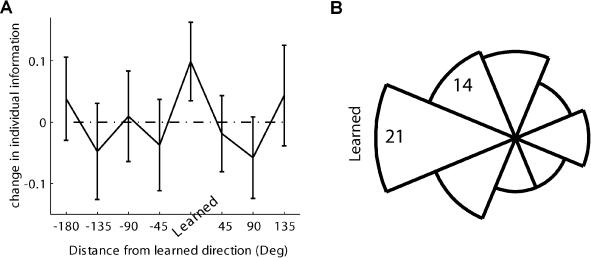
Comparing Individual DI (A) Mean (with 95% confidence intervals, by fitting a Gaussian distribution) of post-learning information minus pre-learning information for one direction. Abscissa represents the distance from the learned-movement direction; all directions were normalized according to the learned direction in the cell's session. An increase is evident only for the learned-movement direction, with mean at 0.1 and 95% confidence intervals at 0.036 and 0.164. (B) Circular histogram of PDs for cells with a positive difference of post-learning minus pre-learning information about the learned direction (Rayleigh test, *p* = 0.01).

We calculated the DI of each cell for each of the eight possible directions, pre- and post-learning. The distribution of the differences between the post-learning DI and pre-learning DI for the learned direction was significantly above zero (Figure 5A, “‘Learned”'). This indicates that after learning, cells' firing rates conveyed more information about the learned direction. Figure 5A also shows that information about other nonlearned directions did not change. As with the MuI, to probe for the directional tuning of the cells, we plotted the circular histogram of PDs of cells that increased their information about the learned direction (a positive post-learning minus pre-learning). Again, a unimodal distribution (Rayleigh test, *p* = 0.01) was found with its peak on the learned direction (Figure 5B).

### Possible Origins for Improvement in Information

Information theory makes use of the complete (estimated from data) stimulus–response distribution and thus does not tell us what feature in cell activity primarily contributed to the increase in information content. However, we found that the increase in information is specific to a single-learned direction and that cells with a PD close to the learned direction mainly contributed to this increase. We have previously reported that cells with PD close to the learned direction increased their firing rate after learning when movement was to the learned direction (Paz et al. 2003). We therefore explored more closely this elevation in firing rates and its relationship to the increase in information content.

**Figure 6:**
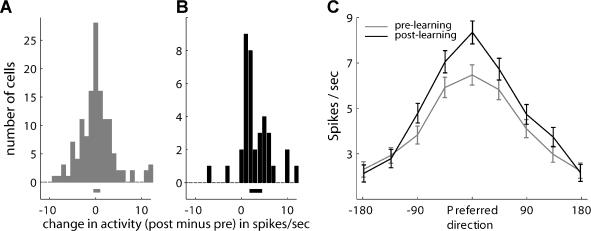
Learning-Induced Elevation of Information and Activity (A and B) Histograms of changes in firing rate in the PD (post-learning minus pre-learning) for all the cells (A) and for cells that significantly increased their information (B). The horizontal line below the histogram represents its mean and the 95% confidence intervals, by fitting a Gaussian distribution. (C) Average tuning curves (baseline subtracted, ± SEM) of cells that significantly increased their information (*n* = 37). Comparing pre-learning (gray) and post-learning (black). Cell tuning curves were first aligned to each cell's PD.

Figure 6A histograms the net changes in activity (post- minus pre-learning) at the cells' PDs for the whole population. Figure 6B shows the same net changes for cells that significantly increased their information about direction, where a significant positive trend was found (by fitting a normal distribution; see legend to Figure 6B). We further aligned each cell tuning curve on the cell's PD and calculated the average tuning curve. This revealed that this group of cells indeed elevated their activity mainly around their PD (Figure 6C).

**Figure 7:**
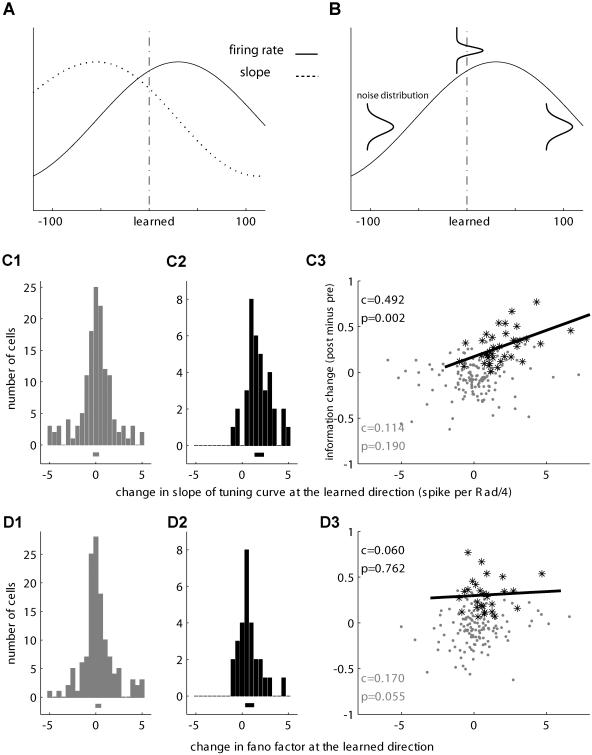
Increased Slope of Tuning Curve Is Correlated with the Increase in Information Possible mechanisms for improving the information content of single cells. (A and C) The slope of the tuning curve at the learned direction indicates the magnitude of change in activity in response to small changes in direction. The higher slope suggests that nearby directions can be discriminated better. (B and D) Reliability of coding. The variability at each direction indicates how well different directions can be differentiated based on single trials. (C1 and D1) Histograms of the difference between pre- and post-learning for the corresponding mechanism for the whole population of cells. The horizontal line below the histogram represents its mean and the 95% confidence intervals. (C2 and D2) Histograms for cells that significantly increased their information about direction. (C3 and D3) Correlation between the difference in information (post- minus pre-learning) and the corresponding mechanism. Gray dots are all the cells, and black asterisks are cells that significantly increased their information content. The black line represents the linear regression fit. The corresponding Pearson correlation (C) and its significance (*p*-value) are designated. The histogram in (C2) is shifted to the right, indicating that cells that increased their information content also increased the slope of the tuning curve in the learned direction. In these cells only, a significant (*p* = 0.002) correlation coefficient (*c* = 0.492) was found.

**Figure 8:**
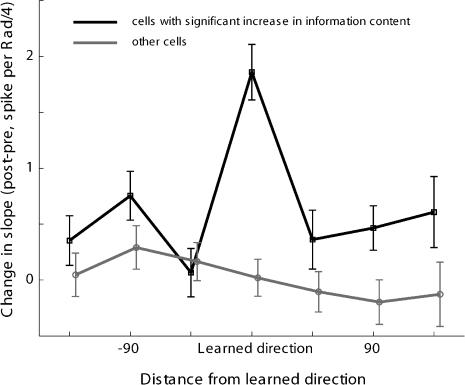
Slope Increase Is Specific to the Learned Direction Mean change (± SEM) in the slope of the tuning curve surrounding each direction, for cells that significantly increased their information content (black) and for the rest of the cells (gray).

Two natural features of a cell's tuning curve can contribute to the improvement in information content. First, a cell can increase the slope of the tuning curve just near the learned direction, and thus small changes in direction can lead to a larger difference in the cell's response, providing a better differentiation of direction based on the neuronal activity (illustrated in Figure 7A). Second, cells can reduce the variability of their response near the learned direction. This is also termed “reliability,” because when variability is low, each single report made by the cell is more reliable (illustrated in Figure 7B). A standard method for characterizing this is the Fano factor (Berry et al. 1997), calculated as the variance of the response divided by its mean. We correlated the net change in information content (post-learning minus pre-learning) to these two factors: change in slope near the learned direction (Figure 7C1–7C3) and change in the Fano factor (Figure 7D1–7D3). Figure 7 shows that whereas no systematic change in the corresponding factor was found for the whole population (Figure 7C1 for slope and Figure 7D1 for FF), a significant positive trend was observed for the population of neurons that significantly increased their information after learning. This trend was obvious for the slope factor (Figure 7C2) and also, but to a much lesser extent, for the Fano factor (Figure 7D2). Figure 7C3 and 7D3 extends this relation and shows the correlation between the corresponding factor and the increase in information. A significant positive correlation was only found for the slope factor and only for cells that significantly increased their information (Figure 7C3, black asterisks and line). No correlation was observed between the change in slope and the change in information for the rest of the cells (Figure 7C3, gray dots) or between the change in Fano factor and the change in information, either for the whole population (Figure 7D3, gray dots) or for those that significantly increased their information (Figure 7D3, black asterisks). Further, the increase in the slope of the tuning curve near the learned direction was specific to this direction only and to cells that significantly increased their information content (Figure 8).

**Figure 9:**
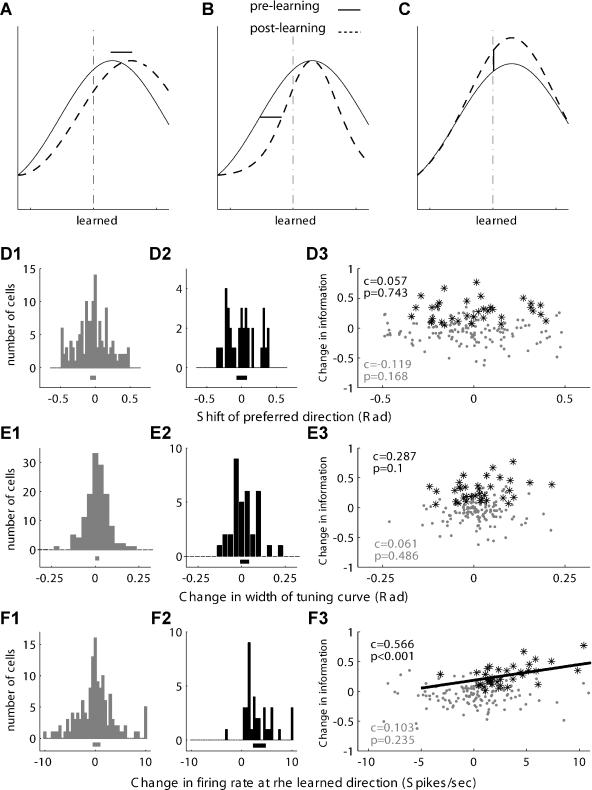
Increased Information after Learning Is Correlated with Elevation of Firing Rate in the Learned Direction Possible mechanisms for increased slope of the tuning curve in the learned direction. (A and D) Shift of PD, i.e., shifting the whole tuning curve, may position the learned direction at a higher slope location. (B and E) Narrowing of the tuning curve, as measured by the width at half-height. (C and F) Local changes (Increase) in activity in the learned direction can increase the slope. This is similar to the observed learning-induced changes in our data (see Figure 6C). In (A)–(C), an illustration of the measured difference is indicated. (D1–D3, E1–E3, and F1–F3) Same format as in Figure 4 for the three possible mechanisms. The histogram in (F2) is shifted to the right, indicating that cells that increased their information content also elevated their firing rate in the learned direction. In these cells only a significant (*p* < 0.001) correlation coefficient (*c* = 0.566) was found (F3, asterisks and line).

These results suggest that cells increased the slope of their tuning curve near the learned direction and improve the information content in their activity. Cells can use several strategies to do so and we considered three possibilities: first, by shifting their tuning curve and positioning the learned direction at a better “slope-wise” location on the tuning curve (illustrated in Figure 9A); second, by narrowing the tuning curve (Figure 9B); and, third, by local changes increasing or decreasing specific points near the desired (learned) location (Figure 9C). Although the three possibilities are not mutually exclusive and might be interrelated, we attempted to distinguish among them by correlating the change in information to each one. Figure 9D–9F shows that the increase in information was correlated with the increased firing rate at the learned direction (Figure 9F1–9F3), but not with shifts in PD (Figure 9D1–9D3) or with the narrowing of tuning curves (Figure 9E1–9E3). We therefore suggest that cells locally increased their firing rate to increase the slope of their tuning curve at the learned direction.

### Decoding Movement Direction

We hypothesized that the improved information regarding movements in the learned-movement direction would lead to an improved ability to reconstruct movements from population activity. To test this assumption, we applied two reconstruction methods: the population vector (PV) approach, a widely used decoding scheme for M1 activity (Georgopoulos et al. 1988; Moran and Schwartz 1999), and a maximum a posteriori (MAP) estimator (Sanger 1996).

**Figure 10:**
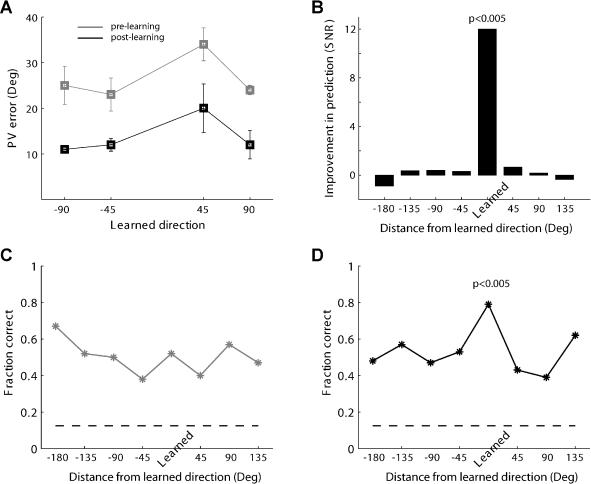
Improved Decoding of Movement Direction Only for the Learned Direction (A and B) Using PV. (A) PV errors given as the distance in degrees between the predicted and the actual direction for the four learned-movement directions (± SEM, bootstrap test). (B) Signal-to-noise ratio (mean/SD) of PV improvement (pre-learning deviation minus post-learning deviation) for all directions (four learned directions are pooled together and all other directions are normalized to them). A significant improvement was observed only for the learned direction (*p* < 0.005, Bonferoni correction for multiple tests, i.e., the eight directions). (C and D) Using a MAP estimator, we predicted 100 times the actual hand direction using neuronal activity. Shown is the fraction of correct predictions for pre-learning (C) and post-learning (D). A significant increase was observed only for the learned direction (*p* < 0.005, Bonferoni correction for multiple tests, i.e., the eight directions). The dashed line is the chance level (0.125).

For the PV analysis, we selected 129 of the 177 cells, only including cells that exhibited directional tuning by a cosine fit. Neurons were pooled according to the learned-movement direction in their recording session, and we computed the PV from the pre-learning and post-learning activity. Figure 10A shows the deviation of the PV direction, i.e., the difference between the PV prediction and the actual movement direction for the four possible learned-movement directions. A marked and statistically significant improvement was observed in the predicted direction (*p* < 0.05 for all four learned directions, bootstrap and *t*-test). We verified that this improvement was due to learning in two ways: first, by shuffling trials from the pre-learning and the post-learning epochs, and second, by shuffling cells from days with different transformations. In both cases, the mean of the distribution of improvements was not significantly different from zero. Furthermore, the improvement in the PV prediction was specific to the learned-movement direction. Figure 10B shows the signal-to-noise ratio (mean/SD) of improvements in PV accuracy (the difference between the accuracy of the pre-learning prediction and the post-learning prediction). We normalized each session directions to the learned direction in the session. A statistically significant improvement was found only for the learned-movement direction (χ^2^, *p* < 0.01). This improvement in the PV prediction can be accounted for by the enhanced firing of cells with a PD near the learned-movement direction, as shown above (see Figure 6). Cells with their PD close to the learned-movement direction made a larger contribution to the PV, but mostly when the movement was in that direction. Because each cell contributes a weighted vector in the direction of its own PD, only the learned-movement directions showed improvement in PV accuracy. This improvement in prediction due to altered directional tuning is reminiscence of studies that examined learning of visuomotor associations in frontal eye fields (Chen and Wise 1996, 1997) and of studies showing evolvement of directional tuning in M1 when monkeys received real-time visual feedback of brain-controlled trajectories (Taylor et al. 2002).

The PV method includes several assumptions about the coding and the decoding of the M1 population activity and is not guaranteed to be optimal (Sanger 1994; Snippe 1996; Pouget et al. 2000; Scott et al. 2001). Therefore, we also tested the performance of a probabilistic approach. Using a MAP estimator, we predicted the movement direction for all possible directions, including the learned-movement direction pooled and normalized from all sessions. Figure 10C depicts the success rate for 100 repetitions (by cross-validation) for each direction. Figure 10D shows the same, but in the post-learning epoch. A higher success rate of correctly predicting the movement direction can be observed for learned direction only in the post-learning epoch (χ^2^, *p* < 0.01, chance level is at 0.125;dashed line in Figure 10D). This indicates that after learning and by using this decoding method, we could better predict the actual movement direction from neuronal activity.

## Discussion

This report describes improved encoding and decoding of specific directions by neurons in M1 of monkeys after learning a visuomotor skill that requires learning only for one direction in space. Our results suggest a close link between properties of neurons, such as directional tuning of cells, and learning a skill that is local in the same parameter, in this case direction, a finding that is concordant with ideas and findings in sensory systems (Zohary et al. 1994; Suga et al. 2002; Sharma et al. 2003). The fact that the increased information we found was associated with an increased slope of the tuning curve, as also reported in a recent visual study (Schoups et al. 2001), further suggests that similar mechanisms may govern neuronal interactions and learning throughout the central nervous system.

The fact that improved information in neuronal activity was evident mainly for the learned direction is in accordance with studies showing confined generalization of learning a sensorimotor skill, one that requires adaptation to directional errors. The width of the behavioral generalization function (i.e., the angular distance from the learned direction where aftereffects could still be observed) was similar for our monkeys (Paz et al. 2003) and in human studies, ranging from 45° (Gandolfo et al. 1996; Krakauer et al. 2000) to 90° (Imamizu et al. 1995; Thoroughman and Shadmehr 2000). The neuronal changes we previously observed occurred mainly for cells with PD within 30° of the learned direction, and the change in slope observed in this study was sharply focused and not seen for directions 45° away from the learned direction (see Figure 8; note, however, that changes in firing rate were wider [see Figure 6]). While narrower primitives reasonably lead to narrower generalization function (Donchin et al. 2003), the exact generalization width depends not only on the primitives' width, but also on the connectivity and the specific model used. These are still largely unknown.

An intriguing result in this study is that learning-related changes were observed and persistent in the post-learning epoch, when performing a standard task that required no transformation. Further, measured kinematics was the same as in the pre-learning epoch. If the improved information can be used, why isn't it? First, our monkeys were trained on a task that did not require improved performance in the standard task after learning, but did encourage them to reserve learning for future use of the same visuomotor task. This is in agreement with our previous report, showing that the monkeys retained the task until the performance of the relearning epoch (i.e., they exhibited immediate recall rather than learning in this second learning epoch), and suggests that the neuronal change should persist but somehow gated. Indeed, everyday behavior shows that we can learn new tasks without interfering with the performance of existing ones. An alternative possibility is that we did not measure the appropriate kinematic variable that was altered and improved due to the neuronal changes. For example, a task that would demand finer directional sensitivity (i.e., angular distance of less than 45°) might show a change in performance after learning.

It is also worth noting that our experiment was performed in a local region in space and was not constrained to a specific posture (Scott and Kalaska 1997) or joint or muscle combination (Scott et al. 2001). Therefore, we cannot conclude that locality and specificity of change in information content are related to external direction of movement. Our results may be consistent with other reference frames as well (Mussa-Ivaldi 1988; Todorov 2000).

One important question is what kind of learning can induce such an increase in information content. Although psychophysics studies have shown that adapting to new kinematics and/or dynamics environments results in the formation of internal representations in the brain (reviewed by Kawato 1999; Wolpert and Ghahramani 2000), changes were also observed after extensive training and mere repetition (Nudo et al. 1996). Moreover, many sensory systems exhibit stimulus-related adaptations (Dragoi et al. 2000; Suga et al. 2002), where repeated presentations of a stimulus induce a change in activity of neurons. To control for this possibility, we conducted sessions with a repetition condition, which entailed a one-target task without angular transformations. Cells recorded in these sessions did not exhibit a change in their information content, and PV analysis produced similar results before and after repetition. An alternative explanation could be attention-related modulations (Spitzer et al. 1988). We discuss elsewhere why this is an unlikely source for the changes we observed (Paz et al. 2003), yet we cannot rule out the possibility that increased attention might lead to similar improvement in information.

MuI measures are more often used in sensory research, describing the information that neurons convey about a presented stimulus, and only few papers have applied such measures to the motor system (e.g., Hatsopoulos et al. 1998). We believe this stems from the fact that in sensory systems, neurons respond to the stimulus, whereas in the motor system, neurons “cause” the movement. In this study, we treated direction of movement as a stimulus to which the neuron responds. This can be justified because MuI is a symmetric measure and the point of view can be reversed; e.g., we can interpret the results as neuronal activity → movement, rather than movement → neuronal activity. More importantly, frontal motor fields, M1 included, are only part of the brain's learning system and project to many brain areas that take part in processing an upcoming movement, such as the basal ganglia and cerebellum (Middleton and Strick 2000). Therefore, M1 activity may be decoded by those areas involved in coplanning of the movement. Moreover, an efference copy of the planned motor command is probably used for error estimation and correction (Wolpert and Ghahramani 2000; Sommer and Wurtz 2002). Indeed, we are aware of our movements before they have actually started (Haggard and Magno 1999). This suggests that when learning new sensorimotor tasks, activity in M1 should not only produce the correct behavior, but also change in a way that enables other brain areas a better readout of the motor command. This will allow more efficient computation and better control of the forthcoming movement.

Although higher information content implies better encoding by neurons, it does not entail better decoding; this is highly dependent on the algorithm used and on the error function introduced. Since our task involved manipulation of movement direction and since real-time prediction of movement trajectory has taken on major interest in recent years (Wessberg et al. 2000; Taylor et al. 2002), we used the discrepancy between the actual movement direction and the predicted one from neuronal activity as the error signal (either categorical, for the MAP, or continuous, for the PV). The MAP method (Sanger 1996; Zhang et al. 1998) is theoretically optimal (Seung and Sompolinsky 1993) and requires fewer assumptions on the tuning-curve shapes and distribution of PDs (Sanger 1994), but requires larger amounts of data to estimate the true distributions (Pouget et al. 2000). The PV method has been shown to be robust in many scenarios (Georgopoulos et al. 1988; Moran and Schwartz 1999) and very useful in predicting hand movement in real time (Taylor et al. 2002). In our experiment, both methods yielded a better prediction of the learned-movement direction during its planning stages and long before its initiation (see also Laubach et al. 2000). Although we cannot determine whether neurons further downstream use this improved information to decode a previous layer of neurons, we believe it is possible. Further, our findings could lead to improved strategies for recovering trajectory information from populations of M1 cells (Wessberg et al. 2000; Serruya et al. 2002; Taylor et al. 2002). The specificity of the learning is of high importance here. The large number of degrees of freedom, the complexity of movements, and the technical difficulties of recording many neurons simultaneously are only starting to be addressed, and a plausible strategy might require learning and practicing specific and essential movements. Our results suggest that this would modify brain activity in a way that would enable earlier and better readout of brain activity from fewer neurons.

## Materials and Methods

### 

The experimental setup and data acquisition procedures are described in detail in Paz et al. (2003). The sampled cells were taken from the same database.

#### Physiological procedures.

Two female rhesus (Macaca mulatta) monkeys (approximately 4.5 kg) were implanted with recording chambers (27 × 27 mm) above both the right and left hemispheres. Animal care and surgical procedures complied with the National Institutes of Health *Guide for the Care and Use of Laboratory Animals* (rev. 1996) and with the Hebrew University guidelines supervised by the Institutional Committee for Animal Care and Use. The monkeys were seated in a dark chamber, and eight microelectrodes were introduced into each hemisphere. The electrode signals were amplified, filtered, and sorted (MCP-PLUS, Alpha-Omega, Nazareth, Israel), and all spike shapes were sampled at 24 KHz. We used a template-based method for real-time isolation of spike shapes (MSD, Alpha-Omega).

Penetration locations were verified by MRI (Biospec Bruker 4.7 Tesla, Bruker BioScences, Billerica, Massachusetts, United States) before recordings. At the end of each session, we examined the activity of neurons evoked by passive manipulation of the limbs and applied intracortical microstimulation (50 ms of 200-μs cathodal pulses at 300 Hz) to evoke movements. Only penetration sites that evoked single-joint shoulder or elbow movement at thresholds of lesser than or equal to 40 μA were used in this study. In one monkey, we also made anatomical observations, to verify the accurate penetration sites relative to the central sulcus.

#### Behavioral paradigm.

Monkeys moved a manipulandum to control the movement of a cursor on a video screen located 50 cm from their torso and eyes with the goal of moving the cursor from a starting point at the center of the screen (origin) to a visual target in a delayed go-signal paradigm; this required the monkey to hold (as verified by hand velocity and EMG) the cursor in the origin circle for a random 750–1,500 ms after the target onset. The disappearance of the origin indicated the go-signal. In each session (day), four consecutive epochs were introduced: (1) pre-learning epoch (more than 100 trials), a standard, eight-target task in which the target direction was randomly chosen from eight possible directions uniformly distributed over the circle; (2) learning epoch (more than 30 trials), a transformed, one-target task in which only one target (upwards, 90°) was presented and a rotational transformation was introduced between the cursor on the screen and the manipulandum; (3) post-learning epoch (more than 100 trials), where the default eight-target task was presented again; and (4) relearning epoch, same as the learning epoch. Rotations were 90°, 45°, –45°, or –90° and were chosen randomly for each session, but fixed for the duration of the learning epoch in a session. Note that learning here is local in direction since only one target direction was introduced during the learning epoch. The term *learned-movement direction* refers to the direction of hand movement needed to bring the cursor to the target for these rotations (thus, there were four possible learned-movement directions in this study: 0°, 45°, 135°, and 180°, associated with the –90°, –45°, +45°, and +90° transforms, respectively). Monkeys were trained for several months with the standard eight-target, task but did not see the transformations before the recordings. To achieve learning on a daily basis during the whole recording period (rather than switching between pre-learned behaviors), a different rotational transformation was randomly chosen for each day from the set of four possible transformations. To observe systematic change in the activity of neurons, the same transformation was repeated (greater than or equal to four repetitions for each transformation and each monkey, on different days).

Note that in this paradigm, the monkeys learn the visuomotor rotation by repeated performance of a single movement (to the learned direction). To test whether the repetition could be responsible for the neuronal changes observed, we conducted control sessions. These sessions (termed “‘repetition”' sessions) consisted of a one-target task without any visuomotor transformation (namely, a standard task to one direction only). We performed 16 such sessions (ten with repeated movements to 90° and six with movements to 180°).

#### Data analysis.

Psychophysics studies have shown that immediately after learning, humans exhibit aftereffects, which is evidence for the formation of an internal representation of the newly acquired skill (Lackner and DiZio 1994; Shadmehr and Mussa-Ivaldi 1994; Kawato 1999). This has been observed in monkeys as well (Paz et al. 2003). To compare neuronal activities for movements with same kinematics, we excluded the first trials (three to five) in the post-learning epoch that exhibited significant aftereffects (measured as the directional deviation at peak velocity from a straight movement and compared to the distribution of deviations before learning). For the remaining trials, we compared velocity profiles, initial direction as a function of time, and actual trajectories to verify that there were similar to the trajectories in the pre-learning epoch (see Figure 1B–1D). We also compared reaction times and perpendicular deviations at peak velocity and endpoint locations. No significant difference was found between the pre- and post-learning in all three groups (*t*-test, *p* > 0.1).

We also verified that learning was the same during the whole recording period. We divided the recording period into two to three consecutive segments and compared (1) learning rates in the learning epochs and (2) aftereffect magnitudes and washout rates in the post-learning epoch (Paz et al. 2003).

To further avoid changes in activity that result from any kinematic or dynamic differences, and since learning-related changes were only observed in activity taken from preparation for movement (before the go-signal), here we only report neuronal activity from this period, i.e., activity during the 600 ms following the target appearance but before the go-signal. We isolated 177 cells (113 from monkey W and 64 from monkey X) based on (1) the lack of significant change in activity during the first-hold period (during which no information was available about the upcoming trial) for the pre-learning epoch versus the post-learning epoch (by Mann-Whitney *U*-test); (2) the results of a one-way ANOVA showing a significant effect for direction; (3) more than five trials per direction both pre- and post-learning. We calculated spike counts in the 600-ms range following the target onset, referred to as the PA. Examining the neurons for changes in PD did not reveal any systematic or significant changes (bootstrap test, three of 177 showed a significant change) and PDs were uniformly distributed (Rayleigh test).

MuI between the direction of the movement and each cell response was calculated by standard methods (Cover and Thomas 1991) using the formula



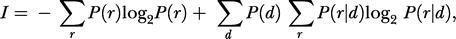



where *d* is the direction of movement and *r* is the number of spikes (see Figure 2). We used either the direct method for calculating *P(r)* or by assuming a Poisson distribution with the mean taken from all trials. We compensated for the limited number of trials (bias correction) by applying either analytical correction (Panzeri and Treves 1996) or by shuffling trials between directions to obtain mean baseline and confidence intervals for the MuI; since both methods produced similar qualitative results, we report here the direct method, corrected analytically.

For calculating the individual DI that each neuron conveys, we used the following formula (Rolls et al. 1997; Buracas et al. 1998) (for alternative definitions, see DeWeese and Meister 1999): 



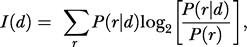



calculated separately for each direction *d*.

To predict the direction of hand movement based on neuronal activity, we used two standard decoding methods:

(1) MAP estimator. This was carried out by assuming Poisson distribution of rates and independency between neurons. We sampled (with repetition) 100 cells and then cross-validated by selecting randomly one trial from each cell and direction, calculating the cell's mean firing rate from the rest of the trials and used the following formula (Sanger 1996) to obtain the most likely direction:



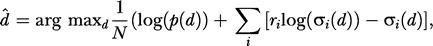



where σ*_i_*(*d*) denotes the mean firing rate of cell *i* in direction *d* and *r_i_* is the rate in the randomly drawn trial. For ease of computation, we took the log of the probability and did not calculate *N*, the normalization factor. The process was repeated 100 times and performed separately for the pre-learning and post-learning.

(2) PV analysis (Georgopoulos et al. 1988; Schwartz 1993). One hundred twenty-nine cells (91 from monkey X and 38 from monkey W) were characterized as directionally tuned by fitting a cosine function (*r^2^* > 0.5). The cells' PDs were homogenously distributed both pre- and post-learning (Rao test, pre-learning, *p* = 0.4, post-learning, *p* = 0.5). We performed two bootstrap tests for significance. First, we shuffled trials from pre- and post-learning and calculated the difference between the deviations of the PV prediction pre-learning to that of the post-learning. The process was repeated 1,000 times to obtain confidence intervals. Second, we shuffled cells from days in which different transformations were learned and again obtained confidence limits. This process tests whether the improvement in prediction was indeed related to the specific direction learned in the session.

## Abbreviations

DI: information per direction
M1: primary motor cortex
MAP: maximum a posteriori
MuI: mutual information
PA: preparatory activity
PD: preferred direction
PV: population vector
